# Thyroid Hormones as Renal Cell Cancer Regulators

**DOI:** 10.1155/2016/1362407

**Published:** 2016-03-13

**Authors:** Łukasz Szymański, Damian Matak, Ewa Bartnik, Cezary Szczylik, Anna M. Czarnecka

**Affiliations:** ^1^Department of Oncology, Military Institute of Medicine, Szaserow 128, 04-141 Warsaw, Poland; ^2^Institute of Genetics and Biotechnology, Faculty of Biology, Warsaw University, Pawinskiego 5a, 02-106 Warsaw, Poland; ^3^Department of Microwave Safety, Military Institute of Hygiene and Epidemiology, Kozielska 4, 01-163 Warsaw, Poland; ^4^School of Molecular Medicine, Medical University of Warsaw, Zwirki i Wigury 61, 02-091 Warsaw, Poland; ^5^Institute of Biochemistry and Biophysics, Pawinskiego 5a, 02-106 Warsaw, Poland

## Abstract

It is known that thyroid hormone is an important regulator of cancer development and metastasis. What is more, changes across the genome, as well as alternative splicing, may affect the activity of the thyroid hormone receptors. Mechanism of action of the thyroid hormone is different in every cancer; therefore in this review thyroid hormone and its receptor are presented as a regulator of renal cell carcinoma.

## 1. Renal Cell Carcinoma

Renal cell carcinoma (RCC) is defined by the National Cancer Institute (NCI), as the most common type of kidney cancer, which develops in the lining of the renal tubules of the kidney [1]. Malignant kidney tumors account for approximately 3% of all malignancies in adults. Men suffer from kidney cancer twice as often as women. The highest incidence of RCC between 1998 and 2006 was observed in North America and the Czech Republic [2]. RCC is a tumor that is resistant to chemotherapy and radiotherapy. Cytokine therapy is beneficial only to a small number of patients (without multiple unfavorable prognostic factors) and is associated with the occurrence of severe adverse effects.

Even though substantial progress in general cancer diagnosis techniques has been achieved, as much as around 20–30% of patients are diagnosed with metastatic RCC (mRCC). What is more, 20% of patients will relapse after nephrectomy and will develop mRCC in the first 12 months after surgery [3]. Molecular therapies such as targeting the mammalian target of rapamycin (mTOR) and vascular endothelial growth factor (VEGF) are the main achievements in modern RCC treatment.

Subtypes of RCC are as follows:

- Clear cell, 83%.
- Papillary (chromophil), 11%.
- Chromophobe, about 4%.
- Collecting duct carcinoma, about 1% [4].

RCC is one of the most vascularized solid cancers and angiogenesis plays a pivotal role in growth of renal tumors (especially ccRCC), because of upregulation of proangiogenic VEGF and platelet-derived growth factor (PDGF). The upregulation is caused by mutation in the von Hippel-Lindau (*VHL*) gene, which induces overexpression of hypoxia-inducible factor (HIF) [3, 5, 6].

The main function of tyrosine kinase is to transfer a phosphate group from ATP to a protein in a cell. Tyrosine kinases function plays a pivotal role in many signal transduction cascades in which extracellular signals are transmitted through the cell membrane to the cytoplasm or nucleus, where gene expression is modulated [7]. Tyrosine kinase receptors (TKRs) are cell surface receptors for many polypeptide growth factors, hormones, and cytokines. TKRs are the key regulators of normal cellular processes and have a critical role in the development and progression of many types of cancer [8]. VEGF and PDGF receptors play crucial roles in angiogenesis, mainly via induction of cell survival, invasion, and proliferation, as well as exhibition of tyrosine kinase activity. To block the physiological function of some of the TKR class receptors, tyrosine kinase inhibitors (TKIs) were developed. Currently the Food and Drug Administration (FDA) approved the following TKIs: sunitinib, sorafenib, axitinib, and pazopanib [5, 9] for use in therapy. Bevacizumab, which is an anti-VEGF monoclonal antibody [3], is commonly used, with clinical benefit. Other new treatment strategies target the mTOR pathway, which is responsible for angiogenesis. Everolimus and temsirolimus are mTOR inhibitors also approved by the FDA [9]. Sunitinib (Sigma-Aldrich) is a tyrosine kinase receptor inhibitor, which targets VEGF-R1, VEGF-R2, VEGF-R3, PDGF-R*α*, PDGF-R*β*, KIT, FLT3, CSF-1R, and RET. Although novel treatment such as using sunitinib significantly prolongs time to progression, the overall survival of patients diagnosed with RCC is still not satisfactory, which justifies the search for new methods and therapy targets, for which it is necessary to understand the mechanisms responsible for the development and progression of this cancer.

## 2. The Thyroid Hormones: Triiodothyronine and Thyroxine

The thyroid hormones (THs), thyroxine (T4) and its active form triiodothyronine (T3), are produced by the thyroid gland. In order for the thyroid gland to produce T3 and T4, iodine is needed. The most common form of TH in blood is T4 (with the ratio T4/T3 = 20/1); however, T4 is converted to four times more potent T3 by 5′-iodinase within the cell. Production of T3 and T4 is regulated via a closed loop feedback—high levels of T3 and T4 in blood plasma inhibit the production of thyroid-stimulating hormone in the pituitary gland. Despite the fact that T3 and T4 are lipophilic, they are not able to diffuse passively through the phospholipid membrane of the cell. Instead, the T3 and T4 hormones are dependent on transmembrane iodothyronine transporters [10, 11].

Thyroid hormones are known for their impact on metabolism, development, and normal growth mostly during fetal development. In adults, deregulation of thyroid hormone levels can cause hypo- and hyperthyroidism, which is connected with a vast number of clinical symptoms [12–15]. What is more, THs may play an important role in tumor promotion or suppression [16].

Triiodothyronine (T3) regulates cell proliferation and differentiation and cell cycle and apoptosis. T3 can act as both an activator and inhibitor of cell proliferation [17]. THs act mainly by their nuclear receptors, which serve as hormone-modulated transcriptional regulators.

Thyroid hormone receptors (TRs) are encoded by two genes, *α* and *β*, which are located on chromosome 17 and chromosome 3, respectively [18, 19]. The* THRβ* gene resides in 3p21-25 chromosomal region, which is known to be a hot spot for mutations in genes involved in RCC pathogenesis [20]. The transcripts of* THRα* and* THRβ* are alternatively spliced to produce three major isoforms of the receptor: TR*α*1, TR*β*1, and TR*β*2. Due to their ability to recruit coactivators and corepressors, those receptors are bipolar in their transcriptional properties, which means that they can activate or repress target gene expression. The chromatin template is modified by auxiliary proteins, which interact with general transcriptional machinery to produce appropriate transcriptional output [21, 22]. Genetic alterations in TRs can cause diseases. Mutations in* THRβ* are usually the cause of resistance to thyroid hormone (RTH) syndrome. Disturbed activity of TRs is also a common phenomenon in human cancers. ccRCC is one of the cancers in which aberrances in TR*β* are frequently observed, due to localization of the gene in the hot spot for mutations [23]. TR*β*1 is a T3 dependent transcription factor. In addition to its metabolic role, TR*β*1 has been known for its tumor-suppressive function [24]. Disrupted expression and activation of TR*β*1 have been previously described in tumors of the thyroid [25], lung [26], breasts [27], or insulinomas [28].

Based on research conducted on the cell lines of breast cancer and hepatocellular carcinoma it was proposed that hypothyroidism accelerates the development of metastases and increases the invasiveness of tumor cells; however, tumor growth is slower. Studies on those cell lines in a mouse cancer model also showed that changes in stromal cells of tumor microenvironment generated in response to low thyroid hormone levels significantly alter the development of cancer [29]. Activation of the kinase-signaling pathway—3-phosphatidyl-inositol (PI3K), serine-threonine protein kinase AKT, and protein kinase B—is associated with a mutation in the TR*β* and the development of cancer [30]. Moreover, the function of TR*β*1 as tumor suppressor indicates that the low expression of the receptor can affect the proliferation of cancer cells [31]. For example, in prostate cancer it was shown that the induction of cells by T3 can increase the proliferation of tumor cells by reducing the B-cell translocation gene 2 (*BTG2*) gene expression which is responsible for the regulation of the G1/S transition in the cell cycle [32].

Research on the role of thyroid hormone and its receptor has proven over the years to be extremely complicated. The role of thyroid hormones and the thyroid hormone receptor is not yet understood. The problem is the fact that the impact of thyroid hormone differs in different types of cancer. Moreover, THs may control many different aspects of the same cancer—metastasis, apoptosis, proliferation, and differentiation—completely differently.

## 3. THR**α** and THR**β** Genes Function in Renal Cancer

**Figure 1:**
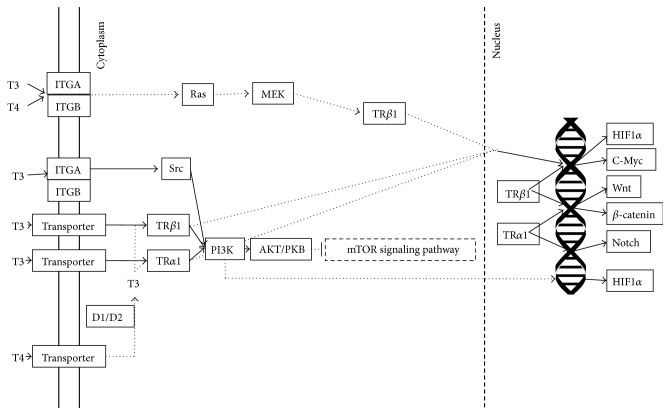
Thyroid hormone-signaling pathway.

TR*β*1 is known for its tumor-suppressive role [24], so it is understandable that expression of this receptor in cancer cell lines is reduced. Reports of the possibility of existence of an alternative thyroid hormone-signaling pathway can be found in literature; Shibusawa et al. [33] researched thyroid hormone action in absence of thyroid hormone receptor. It is known that thyroid hormones are essential for inner ear development; therefore they checked if the same effect occurs in TR*β* ^−/−^ mutant mice. They concluded that although not completely normal, inner ear development could occur in the absence of thyroid hormone receptor; thus it is possible that an alternative and novel thyroid hormone-signaling pathway may exist. Moreover, analysis of the KEGG database showed that thyroid hormones could influence the cell through MAPK and PI3K-AKT signaling pathways, bypassing thyroid hormone receptors as it is shown in Figure 1 [34]. Finally, nongenomic (therefore not-THR mediated) actions of thyroid hormones include the regulation of ion channels, mitochondrial gene transcription, and oxidative phosphorylation [35].

T4 through its own mechanism of action can promote angiogenesis and cell proliferation. Mainly, T4 acts through plasma membrane receptor on integrin *α*v*β*3. *α*v*β*3 is linked with MAP kinase—extracellular signal-regulated kinase (ERK) 1/2—for transduction of the signal initiated by T4 [36]. What is more, Lin et al. proposed a model of action for T3 and T4 at the receptor domain of *α*v*β*3. In this two-site model, site one (S1) is exclusively reserved for T3 and activates PI3K and Src kinase. Activation of those kinases leads to the transcription of HIF-1*α*. Site two (S2) however can be activated by both T3 and T4 and act through ERK1/2 activation to cause tumor cell proliferation [36]. Since signaling pathway activated by T4 via *α*v*β*3 can lead to gene expression, thus, both nongenomic effects of T3 and T4 as well as genomic effects of T3 may overlap in the nucleus [37].

What is more, tetrac which is a deaminated T4 analog has been shown to block hormone binding at S1 and S2 of integrin *α*v*β*3. Tetrac blocks nongenomic action of T3 at S1 and both T3 and T4 action at S2; therefore tetrac suppresses the proliferative nongenomic activity of thyroid hormone on cancer cells [36]. This data is supported by finding of Yalcin et al. who show that tetrac and tetrac nanoparticles reduced tumor volume in human RCC xenografts [38].

Effects of T3 differ between healthy and cancer cell lines. The effect of TH depends on the cell developmental state (stem/progenitor/differentiated) and type and physiological state (cancerous/healthy) [17]. Since in this case healthy and cancer cell lines are compared, it is understandable that the effects of T3 hormone are different. Effect of T3 on the cell cycle in RCC cell lines has never been described previously. According to the literature, effects of T3 can promote or decrease the proliferation rate in different cell lines [17, 39, 40]. T3 is produced by (extrathyroidal) conversion of T4 by DI highly expressed in kidney. The expression of DI has also been reported as downregulated in RCC (in comparison to normal kidney) [41].

## 4. THR**α** and THR**β** Genes Mutations in Renal Cancer

It had been previously shown that the THR*β* gene is frequently mutated in RCC [42–44]; however it was only shown for tissue samples but not established cell lines. In first report Kamiya et al. [42] found that 40.9% of tested ccRCC samples had at least one TR mutation; however, there is no information about the reference sequence or the percentage of mutations in samples derived from the healthy kidney. Nevertheless, mutations were reported to be found in seven of twenty-two investigated cancer samples. Finally, if the ccRCC samples were compared with samples from the healthy kidney, there is no information if sequences obtained from control samples were identical to each other. Future sequencing research with a broader panel of RCC cell lines and with different primers is needed; however, it is possible that commercially available cell lines are an inadequate model to investigate mutations in RCC. THR*β* mutations were further studied functionally and it was shown that most of these RCC mutants result in expression of a receptor that is impaired in T3-mediated transcriptional activity. Moreover, receptors that are not fully functional are dominant-negative inhibitors of wild type* THRβ1* expressed from second allele. Mutations in the T3-binding domains of the receptor—S380F, E299K, H412R, Y321H, and L456S—reduce T3 binding activity at different rate between 35 and 60%. In specific cases including Q252R W219L, F451S, F451I, F417L, and A387P up to 100% of T3 binding activity is lost. Mutations in DNA binding domain—like K155E—may result in the 100% loss of DNA binding activity of thyroid hormone receptor [42]. In selected mutants loss of transcriptional activity results from lower avidity and defective T3 binding. In other cases, transcriptional inefficiency reported for* THRβ1* mutants results from altered corepressors binding and release. These mutants may require significantly higher hormone concentration to release the corepressor and activate transcription. In selected cases increase in corepressor affinity is accompanied with modified corepressor and corepressor splice variants specificity and this may render* THRβ1* mutants toward resistance repression inhibition [45]. Finally,* THRβ1* mutants were shown to harbor greatly expanded target gene specificity that is not homologous with that of the wild type* THRβ1.* Interestingly upregulation of the von Hippel-Lindau (VHL) tumor suppressor was found in rc15-*TRβ1* mutant cells [44]. In functional study T3 had no direct effect on transcription of* HIF-1α,* but activation of* THRβ1*/*RXR*-*α* (retinoid X receptor alpha) heterodimer by T3 stimulated expression of HLF (hepatic leukemia factor), which finally promoted* HIF-1α* expression [46].

## 5. THR**α** and THR**β** Genes Expression in Renal Cancer

Disrupted TR*β*1 activity may be the result of reduced TR*β*1 expression, loss of heterozygosity at the* THRB* locus [47], decreased intracellular concentrations of T3, mutation in DNA at the* THRB* locus [42], altered interaction with coregulators [45], aberrant splicing and sequence of TR*β*1 UTRs, or increased expression of miRNAs that interact with the* THRβ1* 3′ UTR [31]. In first reports* THRβ1* at the transcriptome level (mRNA) was reported as overexpressed in 30% and downregulated in 70% of tumor samples. In the same set of tissues, at the proteome level, expression of* THRβ1* was decreased 1.7 times in tumors tissues when compared to healthy kidney from the same patients. In this report* THRβ1* protein expression was analyzed in nuclear extracts from RCC tumors. It was revealed that decreased* THRβ1* protein level was found in 87% (20/23) cancer samples when compared with normal adjacent tissue from the same kidney.* THRβ1* protein was decreased 1.2 to 16 times depending on case [48]. Another dissonance between the relative amount of* THRβ* mRNA and protein in the cancer cell line when compared to healthy (control) cells was observed by Master et al. [31]. Expression of* THRβ* was correlated with* THRα* in cancer ccRCC tissues, but not in normal kidney samples [48].* THRβ* protein level in cancer tissues was almost nondetectable [48]. In more recent study TR*β*1 mRNA and protein levels were reduced by 70% and 91% [31]. This 70% reduction was reported on* THRβ* mRNA coding sequence, but reduced expression of 5′UTR variants *A* and *F* has been reported in 75% and 62% tumors, respectively [31]. In RCC cell culture model it was shown that weakly folded variant *A* (AY286465.1) of 5′UTR promotes the highest level of* THRβ* expression, while strongly folded variants *F* (AY286470.1) and *F*1 (GQ456950) result in low transcription and translation rates and low* THRβ* expression and in RCC the delicate balance between *A* and *F*/*F*1 variants is disturbed [31]. Reported discordance in TR*β*1 mRNA level and protein along with mutations in* THRβ* 5′UTRs deregulating mRNA stability and transcription rate suggests that* THRβ* expression is regulated mostly by posttranscriptional events along with epigenetic modifications and specific transcription initiation control [31, 45, 49].

## 6. Thyroid Function and Renal Cancer Treatment

Tyrosine kinase inhibitor RCC therapy is associated with development of hypothyroidism in up to 25% of patients as per registered trials (average value for subclinical and clinical hyperthyroidism) [50–52] and up to 100% in selected—Asian—cohorts [53]. It is important to notice that subnormal levels of serum TSH do not always reflect the presence of subclinical hyperthyroidism and in the major part of available literature the definition of hypothyroidism is unclear. Among patients treated with sorafenib, sunitinib, pazopanib, and axitinib, those treated with axitinib develop thyroid dysfunction faster and more pronounced compared to the others [53]. The mechanism through which this tyrosine kinase inhibitor (TKI) causes changes in function of the thyroid is not yet fully understood; however, emerging evidence suggests that sunitinib induces hypothyroidism by altering T4/T3 metabolism and thyroid capillary regression. Sunitinib was found to have antithyroperoxidase activity with potency equivalent to 25% of propylthiouracil (6-propyl-2-sulfanylpyrimidin-4-one). It was found that on sunitinib treatment oxidation of iodide ions to iodine atoms required for the synthesis of T4 is reduced [54]. In cell culture model, sunitinib was shown to induce thyroid cells proliferation inhibition (IC_50_, 14.6 *μ*M) and dose-related increase of (125) I-iodide uptake [55]. Computed tomography volumetry of thyroid gland on sunitinib treatment revealed that more than 50% reduction in volume may develop in half of the patients [56]. Histological study revealed that thyroid atrophy of thyroid follicles with degeneration of follicular epithelial cells is found on autopsy of patients treated with sunitinib for RCC. Surprisingly no decease in vascular volume in the thyroid gland was found in these patients [56].

In clinical research it was also shown that iodine uptake is impaired in RCC patients treated with sunitinib and the lowest uptake is reported at the end of on-drug period of sunitinib treatment (end of 4th week of treatment) [57]. Most recently it was investigated if autoimmunity is coresponsible for thyroid dysfunction in RCC patients treated with sunitinib. It was found that up to 25% of patients develop anti-thyroid peroxidase (TPOAb) autoantibodies within 12–18 month of therapy with sunitinib. Moreover it was found that progression-free survival (PFS) is significantly longer in patients developing these TPOAb in comparison to those who do not develop autoimmune response (10.8 months versus 5.8 months) [58].

There are also a few other possible explanations: TKI-induced inhibition of VEGF receptor tyrosine kinases on thyroid cells that result in capillary regression [59, 60], inhibition of iodine uptake [57], and inhibition of the protein product of the* RET* protooncogene [61]. Moreover, hypothyroidism induced by TKIs such as sunitinib may not be an unwanted toxicity because T3 promotes proliferation of cancer stem cells and metastatic and primary cell lines. Moreover, it was proven that T3 reportedly increased the growth of glioma cells [62]. Schmidinger et al. [63] showed that patients who developed subclinical hypothyroidism during treatment with sunitinib had a significantly greater probability of responding to treatment.

Although correlations between hypothyroidism and treatment outcome have been noticed [64] and many researchers confirmed an association between progression-free survival (PFS) and hypothyroidism [65], the rate of objective remission (ORR) (hypothyroid patients versus euthyroid patients: 46.7 versus 13.7%, resp.; *P*  =  0.001), and the median overall survival (hypothyroid patients versus euthyroid patients: 39 versus 20 months, resp.; *P*  =  0.019) [66], some researchers argue that there is no certainty that hypothyroidism can be considered as an independent prognostic marker, because it is still unclear if the induction of hypothyroidism is the mode of sunitinib action or this phenomenon is dependent on pharmacokinetics, height, and weight of the patient or affinity to tyrosine kinase receptor [63]. It was suggested that although PFS is longer in these patients, there might be no difference in overall survival of hypothyroid patients [67].

Finally, in their study Schmidinger et al. [63] reported that there is a statistically significant correlation between hypothyroidism during the treatment of kidney cancer using tyrosine kinase inhibitors and the percentage remission (patients with hypothyroidism versus euthyroid patients: 28.3% versus 3.3%, resp.; *P* < 0.001) and the mean survival time (above and below 13.9 months, resp.; *P* = 0.016). In another study hypothyroidism was associated with a longer PFS in patients treated with sunitinib or sorafenib (16.0 months versus 6.0 months) and replacement with l-thyroxine did not show influence on PFS [64]. Recent meta-analysis that covered 250 patients has shown that there is no significant statistical difference in PFS between patients with hypothyroidism on sunitinib and patients without acquired hypothyroidism (HR = 0.82 and *P* = 0.22). Similar result was calculated for OS (HR = 0.52 and *P* = 0.01). This leads the authors to the conclusion that development of hypothyroidism during tyrosine kinase inhibitor therapy may not be an independent predictive factor in patients with metastatic renal cell cancer [68].

## 7. Conclusion and Future Perspectives

It is known for a long time that T3 has high impact on cancer development and maintenance; however, there is no fundamental and comprehensive analysis of the role of thyroid hormone in different populations of cancer. Of course, many research groups study T3 influence on cancer; however those findings are not structured and cannot be compared to each other, because thyroid hormone can act differently in different types of cancer. Many research groups provided very important data about T3 and cancer; for example, it has been shown by Poplawski and Nauman that T3 promotes cell proliferation in RCC* in vitro* but does not have the same effect on a cell line derived from healthy kidney [69]. What is more, Puzianowska-Kuznicka et al. [48] and Master et al. [31] observed that cells derived from RCC have disrupted TR*β* expression on both the mRNA and protein level; therefore disrupted posttranscriptional regulation has been proven for RCC. Research by Kamiya et al. [42] showed numerous mutations in the THR*β* transcript in the samples derived from RCC patients. However, it is important to mention that studies cited in this paper used supraphysiologic concentrations of T3 in* in vitro* experiments. Concentration of T3 in human sera was described in numerus studies by many groups as 3.07 nM [70], 1.92 nM [71], 1.69 nM [72], and 5.07 nM [73]. Therefore, it is important to conduct an experiment with physiological concentrations of T3. What is more, based on these and many other publications it is possible to design and perform functional analysis of the TR*β*1 in different populations of cancer, representing primary tumor, metastatic tumor, and cancer stem cell populations of RCC. It is extremely important to understand the mechanisms responsible for the disrupted influence of T3 hormone on cells of renal cell carcinoma because of its role as a cancer regulator and its link to therapy with sunitinib and other multitargeted receptor tyrosine kinase inhibitors.
